# Vertical differentiation of soil properties and microbial communities in long-term *Eucalyptus grandis* × *urophylla* plantations: surface diversity recovery does not offset deep-layer functional degradation

**DOI:** 10.3389/fmicb.2026.1896384

**Published:** 2026-08-05

**Authors:** Zongsheng Yuan, Sifan Wang, Youxian Chen, Xun Dong, Fang Liu

**Affiliations:** 1 Fujian Key Laboratory on Conservation and Sustainable Utilization of Marine Biodiversity, College of Geography and Oceanography, Minjiang University, Fuzhou, China; 2 College of Life Sciences, Fujian Agriculture and Forestry University, Fuzhou, China

**Keywords:** *Eucalyptus grandis* × *urophylla*, long-term plantation, soil aggregate, soil degradation, soil microbial diversity

## Abstract

**Aims:**

Long-term *Eucalyptus* cultivation causes soil degradation, yet the vertical stratification of soil physicochemical properties and its impact on the vertical succession of microbial communities remain poorly understood. This study aimed to clarify the effects of long-term *Eucalyptus* planting on the vertical succession of soil properties and microbial communities.

**Methods:**

This study investigated first-rotation (5-year, E1) and third-rotation (21-year, E3) *E. grandis* × *urophylla* plantations, with mixed *Cunninghamia lanceolata* and *Pinus massoniana* forest serving as the control (CK). Soil samples from 0 to 20 cm, 20–40 cm and 40–60 cm layers were collected to explore vertical variations in soil physicochemical properties, enzyme activities and microbial communities.

**Results:**

Total potassium (TK) content was significantly higher in the third-rotation plantation (E3) than in the control (CK), and increased with soil depth across all plantation types. Available phosphorus and organic carbon first rose briefly then declined overall, and both decreased with soil depth. Urease, acid phosphatase and sucrase activities declined obviously with stand aging and increasing soil depth. Bacterial α-diversity declined initially, then recovered and reached its peak in the E3 topsoil. Microbial community structure changed greatly; dominant microbes showed distinct age- and depth-dependent distribution patterns. Plantation age positively affected soil pH and total potassium but negatively reduced aggregate stability.

**Conclusion:**

Successive *Eucalyptus* planting altered soil physicochemical and enzymatic profiles, and reshaped the vertical microbial community structure.

## 1 Introduction

*Eucalyptus* plantations represent a major plantation forest type in tropical and subtropical regions and have been widely established because of their rapid growth, short rotation cycles, and high economic returns (Pierre et al., 2022). However, with the continued expansion of multi-rotation cultivation, intensive management practices, and short-rotation production systems, soil degradation in *Eucalyptus* plantations has become increasingly severe and has emerged as a major constraint on ecological sustainability (Ma et al., 2022). Soil degradation resulting from long-term *Eucalyptus* cultivation causes pronounced deterioration in soil physicochemical properties across depths, mainly including aggravated soil acidification, reductions in soil organic carbon and total nitrogen contents, and imbalances in phosphorus concentrations (He et al., 2025; Nunes et al., 2024). Notably, these changes show clear soil-depth dependence: surface soils, which are more directly affected by litter input and root activity, exhibit much greater variation in nutrient availability and physicochemical properties than deeper soils (Dai et al., 2023), whereas deeper soils may serve as sources of essential nutrients under specific conditions, and their functional status was closely related to plantation age, management intensity, and root distribution patterns (Lu et al., 2026). This depth-dependent heterogeneity in soil physicochemical properties not only reshapes the spatial distribution of nutrients (Jiang et al., 2024) but also provides an important basis for further investigation of the differentiated responses of microbial communities.

Soil microbial communities play an indispensable role in regulating key processes in plantation ecosystems, including nutrient cycling, organic matter decomposition, and carbon sequestration (Zhang et al., 2026). Long-term Eucalyptus cultivation alters soil physicochemical properties, particularly through depth-dependent changes, thereby reshaping the structure, diversity, co-occurrence networks, and ecological functions of microbial communities (Wang et al., 2025). It also markedly changes soil microbial community composition by decreasing the relative abundance of key functional groups and increasing the proportion of stress-tolerant or potentially pathogenic taxa (Xu et al., 2022). Although previous studies have examined the effects of plantation age, litter management, rotation cycles (Sertutxa et al., 2024), and land-use change (Xu et al., 2022) on soil properties in Eucalyptus plantations, and numerous studies have characterized soil physicochemical properties and microbial communities across different soil depths, a systematic understanding of the integrated causal pathway linking long-term Eucalyptus cultivation, depth-dependent differentiation of soil properties, vertical restructuring of microbial communities, and progressive soil degradation remains lacking. In particular, how depth-specific changes in soil properties jointly drive the vertical succession of microbial communities, and whether these microbial shifts in turn mediate the intensification of soil degradation across depths, has not been disentangled. Furthermore, it remains unclear how depth-related shifts in functional microbial groups affect nutrient cycling and soil degradation, and the underlying mechanisms are yet to be resolved.

Therefore, this study focused on *Eucalyptus grandis × urophylla* plantations aged 5 and 21 years, with an adjacent mixed forest of *Cunninghamia lanceolata* and *Pinus massoniana* serving as the control (CK). Through the analysis of multidimensional indicators encompassing soil properties and soil microbial communities, the pathways driving soil functional changes under long-term *E. grandis × urophylla* cultivation were clarified, thereby providing a theoretical basis for the sustainable management of *Eucalyptus* plantations. The following questions were addressed: (1) How does long-term cultivation of *E. grandis × urophylla* drive the differentiated evolution of soil physicochemical properties across different depths? (2) How does depth-dependent differentiation of soil physicochemical properties reshape microbial community structure and diversity, and what mechanisms regulate these interactions?

## 2 Materials and methods

### 2.1 Study area and plot design

This study was conducted in 2025 at the Tianma State-owned Forest Farm in Zhangzhou City, Fujian Province, China, where *E. grandis × urophylla* plantations were selected as the study area. This region is one of the main cultivation zones for *Eucalyptus* plantations in China. The study area is characterized by a subtropical monsoon climate with warm and humid conditions, distinct wet and dry seasons, a mean annual temperature of 21.5 °C, a mean annual precipitation of 1,500–1,700 mm, annual evaporation of 1,100–1,200 mm, abundant sunshine, and a relatively long frost-free period. The area has a mean elevation of 300–600 m and a mean slope of 15°–20°, with red soil as the dominant soil type and laterite occurring locally.

**FIGURE 1:**
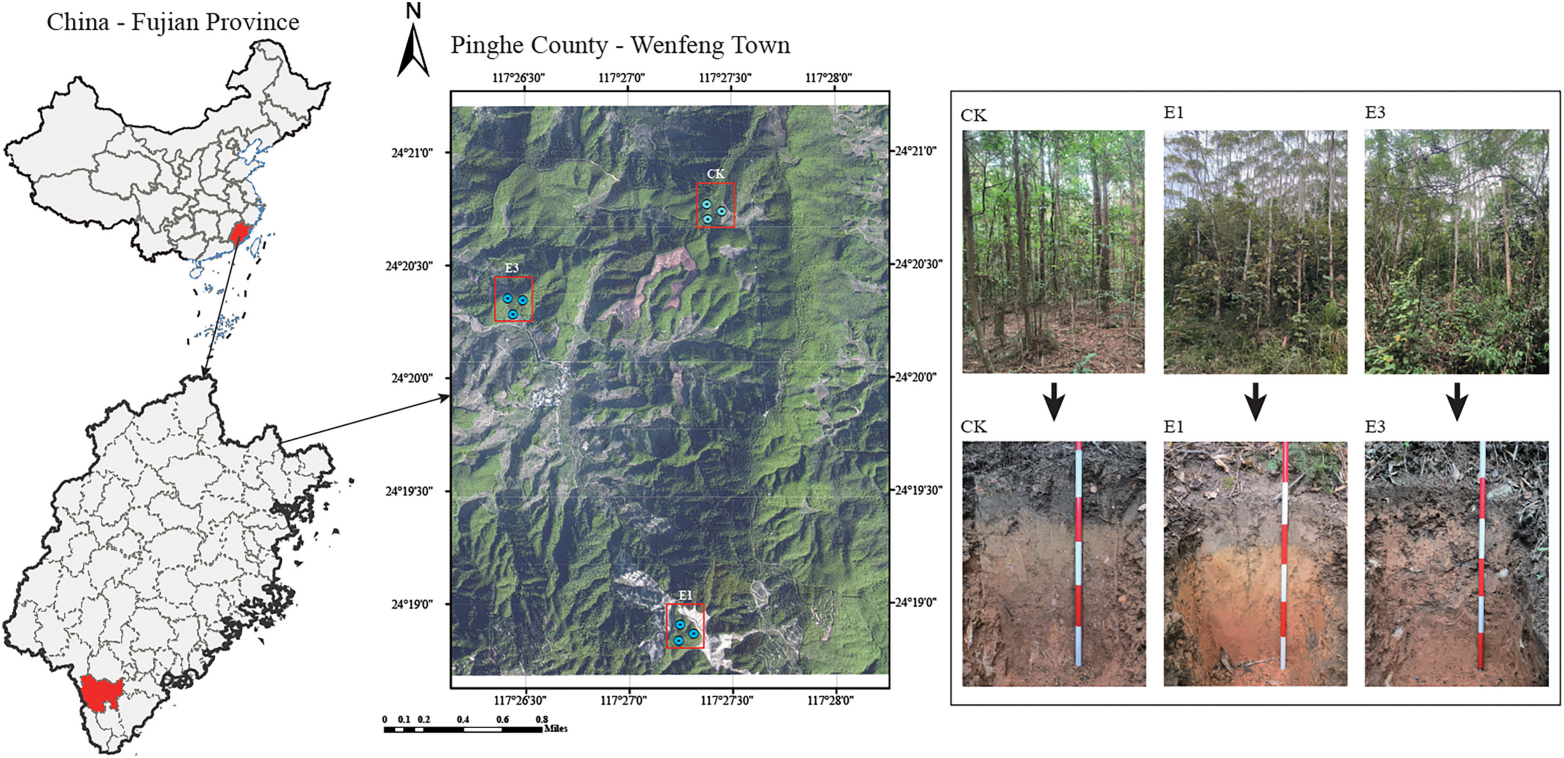
Schematic overview of the study plots.

Monoculture *E. grandis × urophylla* plantations were selected as the research subject, and two plantation plots representing different plantation ages were established: a 5-year-old *E. grandis × urophylla* plantation (first rotation, E1) and a 21-year-old *E. grandis × urophylla* plantation (third rotation, E3), both of which were planted in 2021. Before the cultivation of *E. grandis × urophylla*, both plots were occupied by mixed forests of *Cunninghamia lanceolata* and *Pinus massoniana*, which were harvested and cleared before planting. During the first year after planting, compound fertilizer (N:P_2_O_5_: K_2_O = 15:15:15) was applied as a basal dressing at a rate of 0.25 kg per plant, followed by a single top-dressing application of the same fertilizer at the same dosage 6 months later. Thereafter, all stand management activities, including fertilization and manual weeding, were discontinued, and the stands were allowed to develop naturally. An adjacent mixed forest of *C. lanceolata* and *P. massoniana* was used as the CK. The CK stand was a natural-origin forest established in 1979 (currently 47 years old as of 2026), which has remained unfertilized and unharvested since its establishment. Additionally, all stands shared similar topographic characteristics, including aspect and slope gradient, as well as soil type. Additional details on plot characteristics are provided in Supplementary Table 1, and a general overview of the study plots is shown in Figure 1.

### 2.2 Plot survey

A 20 m × 20 m standard quadrat was established in each plot to measure stand structural parameters, including canopy closure, diameter at breast height, tree height, and crown width (Supplementary Table 1). Based on these parameters, three representative trees were randomly selected from each plot. A 1 m-deep soil profile was excavated at a point 1 m from the base of each representative tree on the downslope side, and soil samples were collected from depths of 0–20 cm (surface layer, D1), 20–40 cm (middle layer, D2), and 40–60 cm (deep layer, D3). Sample designations are listed in Table 1. Soil sampling depths were determined according to the natural horizon differentiation observed in each soil profile, with vertical intervals guided by a red-and-white graduated ruler (each color segment = 20 cm) inserted into the profile face (Figure 1). The uppermost 0–20 cm layer corresponded to the humus-rich surface horizon, 20–40 cm to the eluviation/transition horizon, and 40–60 cm to the illuviation or parent material-affected deeper horizon. This depth partitioning reflected consistent visible changes in soil color, texture, and root density across the profiles.

**TABLE 1:** Group and corresponding number.

Group		Group description	Code
Sample plot	Depth		
CK	D1	Mixed forest of *C. lanceolata and P. massoniana*, 0–20 cm soil layer	CK_D1
	D2	Mixed forest of *C. lanceolata and P. massoniana*, 20–40 cm soil layer	CK_D2
	D3	Mixed forest of *C. lanceolata and P. massoniana*, 40–60 cm soil layer	CK_D3
E1	D1	First rotation plantation of *E. grandis × urophylla*, 0–20 cm soil layer	E1_D1
	D2	First rotation plantation of *E. grandis × urophylla*, 20–40 cm soil layer	E1_D2
	D3	First rotation plantation of *E. grandis × urophylla*, 40–60 cm soil layer	E1_D3
E3	D1	Third rotation plantation of *E. grandis × urophylla*, 0–20 cm soil layer	E3_D1
	D2	Third rotation plantation of *E. grandis × urophylla*, 20–40 cm soil layer	E3_D2
	D3	Third rotation plantation of *E. grandis × urophylla*, 40–60 cm soil layer	E3_D3

### 2.3 Sample collection

For each soil layer (D1, D2, D3) of every representative tree, three soil cores were collected horizontally at 10 cm intervals along the profile wall using a cutting ring (100 cm^3^) for bulk density determination, and the surrounding soil was simultaneously collected into sterile zip-lock bags. The three bagged soil samples from the same soil layer were thoroughly homogenized to form one composite sample. Each composite sample was then divided into four portions: (1) for the determination of physical properties; (2) air-dried at room temperature, ground, and passed through a 100-mesh sieve for the determination of chemical properties; (3) stored at 4 °C for enzyme activity analysis; and (4) stored at −80 °C for soil DNA extraction. Bulk density was determined separately for each cutting ring sample, and the resulting values were averaged, with no mixing performed among cores.

### 2.4 Determination of soil physicochemical properties

Physical property determination (Lutenegger, 2022): Soil bulk density was determined using the cutting ring method. Undisturbed soil samples collected with 100 cm^3^ cutting rings were oven-dried at 105 °C to constant weight, and dry soil mass per unit volume was calculated (Equation 1). Total porosity was calculated from the relationship between soil bulk density and soil particle density, the latter being set at 2.65 g⋅cm^−3^ (Equation 2). Soil aggregate composition was determined using combined dry-sieving and wet-sieving procedures. Air-dried undisturbed soil samples were passed through a nested series of sieves with mesh sizes of 5 mm, 2 mm, 1 mm, 0.5 mm, and 0.25 mm for dry sieving, and the mass fraction of aggregates within each size class was calculated. The soil was then reconstituted according to the proportions obtained from dry sieving and was subjected to wet sieving to isolate water-stable aggregates > 0.25 mm, after which their content and mean weight diameter were calculated. All physical property measurements were performed in triplicate, and the results are expressed as mean ± standard deviation.


$$\mathrm{Soil}\,\mathrm{bulk}\,\mathrm{density}\,\left(g/{\mathrm{cm}}^{3}\right)=\frac{\left(m_{2}-m_{1}\right)\times 1000}{V\times \left(1000+W\right)}$$ (1)

In the formula:

*m*_2_ – Ring knife and wet soil mass, unit: grams (g);

*m*_1_ – Ring knife mass, unit: grams (g);

*V* – Ring knife volume, unit: cubic centimeters (cm^3^), *V* = π*r*^2^*h*, where *r* is the inner radius of the ring knife’s edge end (cm), and *h* is the height of the ring knife;

*W* – Soil moisture content, unit: grams per kilogram (g/kg).

The measurement results are expressed as the arithmetic mean, with two decimal places.


$$\mathrm{Total}\mathrm{soil}\mathrm{porosity}\left(\%\right)=\left(1-\frac{\mathrm{soil}\,\mathrm{bulk}\,\mathrm{density}}{\mathrm{Soil}\,\mathrm{particle}\,\mathrm{density}}\right)\times 100$$ (2)

Chemical property determination (Gu et al., 2019; Liu et al., 2024): Total phosphorus (TP) was extracted using the HClO_4_-H_2_SO_4_ digestion method, and available phosphorus (AP) was extracted using the Bray-1 method, and TP and AP were determined using a SAN++ continuous flow analyzer (Skalar, the Netherlands). Available potassium (AK) was extracted with 1.0 mol L^−1^ CH_3_COONH_4_ solution and was measured using a PinAAcle 900H flame atomic absorption spectrometer (PerkinElmer, USA). Soil samples were pretreated with 0.5 mol L^−1^ HCl to remove inorganic carbonates, after which nitrate nitrogen (NO^–^_3_-N) was determined by the nitrosalicylic acid colorimetric method. Total nitrogen (TN) and total potassium (TK) were extracted by HNO_3_-HF-HCl digestion and measured using an inductively coupled plasma optical emission spectrometer (AVIO 200). Organic carbon (OC) was determined by the potassium dichromate-sulfuric acid oxidation method. All chemical property measurements were performed in triplicate, and the results are expressed as mean ± standard deviation.

Soil enzyme activity: Soil urease (S-UE) activity was determined by the indophenol blue colorimetric method (Guo et al., 2012), soil acid phosphatase (S-ACP) activity was measured by the phenol colorimetric method (Hou et al., 2020), and soil sucrase (S-SC) activity was determined by the 3,5-dinitrosalicylic acid colorimetric method (Hou et al., 2020). All enzyme activity measurements were performed in triplicate, and the results are expressed as mean ± standard deviation.

### 2.5 Soil total DNA extraction and sequencing

A 0.5 g soil sample was accurately weighed, and total soil DNA was extracted using the E.Z.N.A.^®^ Soil DNA Kit (Omega Bio-tek, USA). After verifying DNA quality (total amount ≥15 μg, concentration ≥50 ng μL^−1^), the V3–V4 hypervariable regions of the bacterial 16S rRNA gene were amplified using the universal bacterial primers 338F (5′-ACTCCTACGGGAGGCAGCAG-3′) and 806R (5′-GGACTACHVGGGTWTCTAAT-3′). The fungal ITS1 region (between the 18S rRNA and 5.8S rRNA genes) and ITS2 region (between the 5.8S rRNA and 28S rRNA genes) were amplified using the universal fungal primers ITS1 (5′-TCCGTAGGTGAACCTGCGG-3′) and ITS2 (5′-GCTGCGTTCTTCATCGATGC-3′).

The PCR reaction was performed in a total volume of 50 μL, containing 5 μL of 10 × PCR buffer, 5 μL of 2 mmol⋅L^−1^ dNTPs, 1 μL of DNA polymerase, 1.5 μL of each forward and reverse primer, 50 ng of DNA template, and ddH2O to volume. The PCR program was set as follows: pre-denaturation at 94 °C for 10 min; 35 cycles of denaturation at 94 °C for 30 s, annealing at 56 °C for 30 s, and extension at 72 °C for 30 s; and a final extension at 72 °C for 10 min. Each sample was analyzed in triplicate, and no-template negative controls were included.

PCR products were quantified and quality-checked using the QuantiFluor™-ST blue fluorescence quantitative system (Promega, USA). Qualified amplicons were sequenced on the Illumina MiSeq platform at Shanghai Majorbio Bio-pharm Technology Co., Ltd.

### 2.6 Data processing and statistical analysis

Excel 2019 and SPSS 23.0 were used for data preprocessing, as well as analysis of variance (ANOVA) and post hoc tests (MANOVA + Fisher’s LSD, *p* < 0.05) for soil physicochemical properties.

Paired-end reads were merged using FLASH v1.2.11 to generate full-length gene sequences (Magoč and Salzberg, 2011). Amplicon sequence variants (ASVs) were clustered and taxonomically annotated using the RDP Classifier v2.13 (Wang et al., 2007). The number of ASVs and microbial relative abundance were calculated using USEARCH v11 (Edgar, 2010), and absolute abundance tables were generated using QIIME v1.9.1.

Principal component analysis (PCA) based on ASV profiles was performed using the *vegan* package in R v4.1.3 (FactoMineR method). Prior to PCA, ASV abundance data were Hellinger-transformed to reduce the influence of rare taxa and double-zero effects, and the resulting transformed matrix was centered and scaled and visualized with the *ggplot2* v3.3.3 package (Warton et al., 2012). Spearman correlation analysis was conducted using the *stats* package in R v3.6.3 and was visualized with the *pheatmap* package.

Functional profiles of bacterial communities were predicted using FAPROTAX v1.2.1, and fungal functional guilds were assigned using FUNGuild v1.0. Mantel tests (*p* < 0.05) were performed using the linkET v0.0.7.4 package in R v4.2.3 and were visualized with ggplot2 v3.3.3. Differentially abundant ASVs between pairwise comparisons at each soil depth were identified using DESeq2 with thresholds of |log_2_(fold change)| > 1 and adjusted *p* < 0.01. From these pairwise comparisons, ASVs that were significantly downregulated in CK but significantly upregulated in both E1 and E3 were retained as candidate core ASVs. The candidate ASVs were then incorporated into microbial co-occurrence networks constructed based on Spearman correlations (| r| > 0.6, *p* < 0.05). Zi-Pi analysis was performed using the psrl package to classify nodes based on their topological roles within the network. ASVs with Zi > 2.5 (module hubs) or Pi > 0.62 (connectors) were identified as keystone taxa. Structural equation models (SEMs) were fitted using the piecewiseSEM v2.3.1 package in R v4.5.1. Given the categorical nature of plantation age (E1 vs. E3, coded as a two-level factor with E1 as reference) and soil depth (D1, D2, D3, coded as an ordered factor), all predictor variables were treated as categorical factors in the segmented linear mixed models. Due to the limited number of levels (*n* = 2 for age, *n* = 3 for depth), the estimated path coefficients were interpreted as comparisons between category means rather than as per-unit linear effects. Shipley’s d-separation test was used to evaluate overall model fit, with a non-significant Fisher’s *C* value (*p* > 0.05) indicating adequate fit.

## 3 Results

### 3.1 Changes in soil physicochemical properties across different planting years of *E. grandis × urophylla*

**FIGURE 2:**
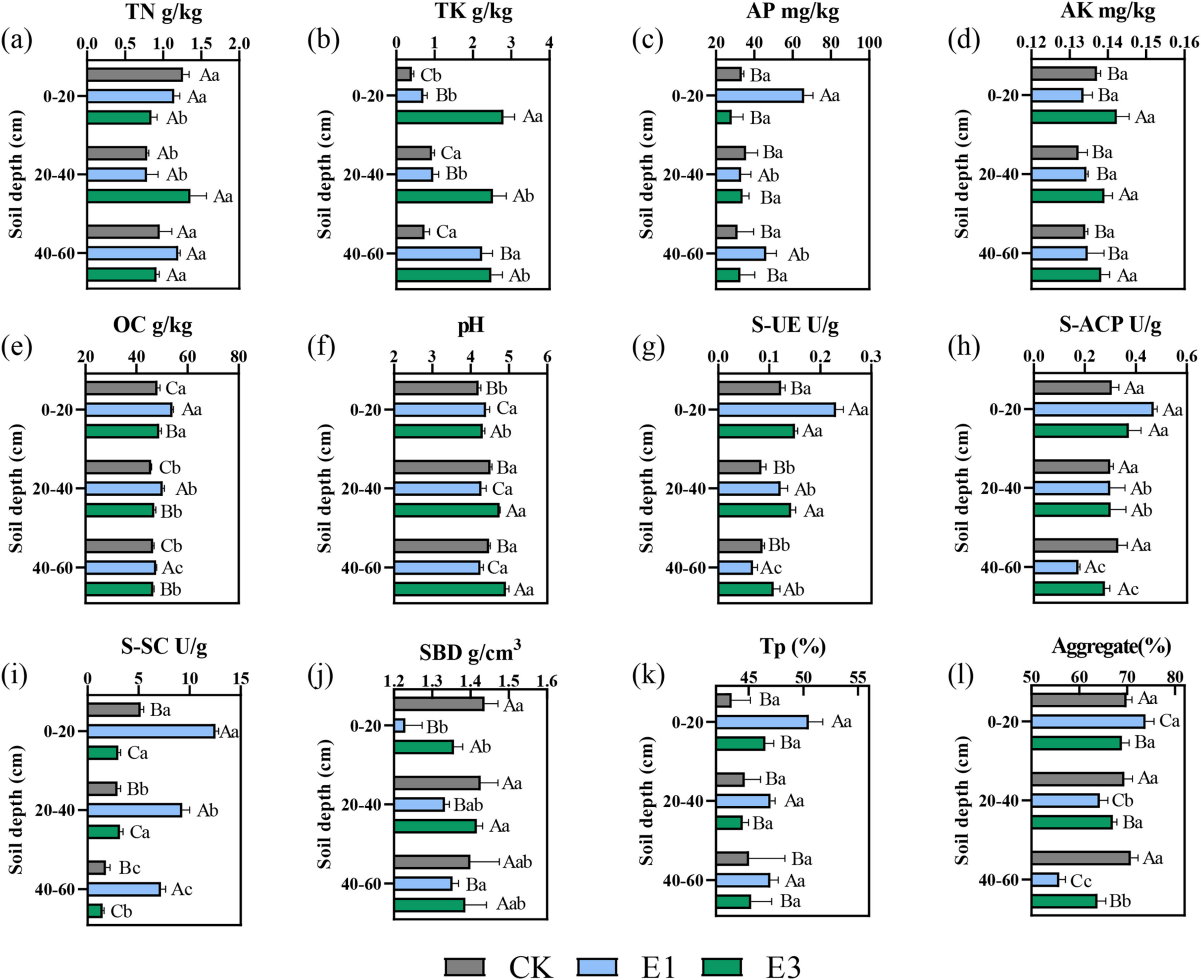
Changes in the physicochemical properties of surface, middle, and deep soil layers across different plantation ages. **(a)** TN, **(b)** TK, **(c)** AP, **(d)** AK, **(e)** OC, **(f)** pH, **(g)** S-UE, **(h)** S-ACP, **(i)** S-SC, **(j)** soil bulk density, **(k)** total soil porosity, and **(l)** soil aggregates. Different uppercase letters indicate significant differences among plantation age groups at the same soil depth, whereas different lowercase letters indicate significant differences among soil-depth groups within the same plantation age (*p* < 0.05).

Compared with CK, E1_D1 showed higher AP (46.25 mg/kg), whereas E1_D3 exhibited higher OC (54.10 g/kg) and S-SC (12.53 mg/g). Additionally, S-UE (0.23 mg/g) and alkaline phosphatase (0.47 mg/g) in E1_D3 were both higher than those in E1_D1. TK (2.80 g/kg) and AK (0.14 g/kg) in E3_D3 were higher than those in CK, and pH reached its highest value in E3_D3 (4.92), whereas the overall values of each indicator in CK were lower than those in E1 and E3. OC in E1_D3 (54.10 g/kg) was higher than that in E1_D1 (47.74 g/kg), whereas bulk density in E1_D3 (1.23 g/cm^3^) was lower than that in E1_D1 (1.35 g/cm^3^). TK in E3_D3 (2.80 g/kg) was higher than that in E3_D1 (0.85 g/kg) (Figure 2). Multivariate analysis of variance (Supplementary Table 2) indicated that both planting years and soil depth had significant overall effects on soil properties. The number of planting rotations (F = 890.76, *p* < 0.01), soil depth (F = 240.15, *p* < 0.01), and their interaction also reached significance (F = 35.50, *p* < 0.001).

#### 3.1.1 Effects of plantation age on soil properties

Compared with CK, the TK content in E3 (2.60 g/kg) was 3.77-fold higher and was significantly greater than that in the first-rotation plantation E1 (1.31 g/kg) (*p* < 0.05). The AP content in E1 (48.50 mg/kg) was significantly higher than that in CK (33.49 mg/kg) (*p* < 0.05). The OC content in E1 (50.69 g/kg) was significantly higher than that in both CK (46.85 g/kg) and E3 (47.50 g/kg) (*p* < 0.05). Soil pH in E3 (4.66) was significantly higher than that in CK (4.40) and E1 (4.31) (*p* < 0.05). Soil bulk density in E1 (1.31 g/cm^3^) was significantly lower than that in CK (1.42 g/cm^3^) and E3 (1.39 g/cm^3^) (*p* < 0.05). Total porosity in E1 (48.15%) was significantly higher than that in CK (44.37%) (*p* < 0.05). Aggregate content in CK (70.04%) was significantly higher than that in E1 (64.66%) and E3 (66.58%) (*p* < 0.05). S-UE activity in both E1 (0.14 mg/g) and E3 (0.13 mg/g) was significantly higher than that in CK (0.10 mg/g) (*p* < 0.05).

### 3.2 Variations in soil properties across different soil depths

Significant differences in TK content were observed across soil depths, with D3 (1.82 g/kg) being significantly higher than D1 (1.30 g/kg) (*p* < 0.05). AP was significantly higher in D1 (42.60 mg/kg) than in D2 (34.31 mg/kg) (*p* < 0.05). OC in D1 (50.44 g/kg) was significantly higher than that in D3 (46.95 g/kg) (*p* < 0.05). Soil pH values in both D2 (4.52) and D3 (4.55) were significantly higher than those in the surface layer D1 (4.31) (*p* < 0.05). Soil bulk density in D1 (1.34 g/cm^3^) was significantly lower than that in D2 (1.39 g/cm^3^) (*p* < 0.05). The aggregate content decreased with increasing soil depth, and D3 (63.47%) was significantly lower than D1 (70.89%) (*p* < 0.05). Regarding enzyme activities, S-UE, S-ACP, and S-SC were all significantly higher in the surface layer than in the deeper layers: S-UE activity in D1 (0.17 mg/g) was significantly higher than that in D3 (0.09 mg/g) (*p* < 0.05); S-ACP activity in D1 (0.38 mg/g) was significantly higher than that in D3 (0.26 mg/g) (*p* < 0.01); and S-SC activity in D1 (6.92 mg/g) was significantly higher than that in D3 (3.48 mg/g) (*p* < 0.05).

### 3.3 Changes in soil microbial alpha diversity across different plantation ages of *E. grandis × urophylla* and identification of key environmental drivers

**FIGURE 3:**
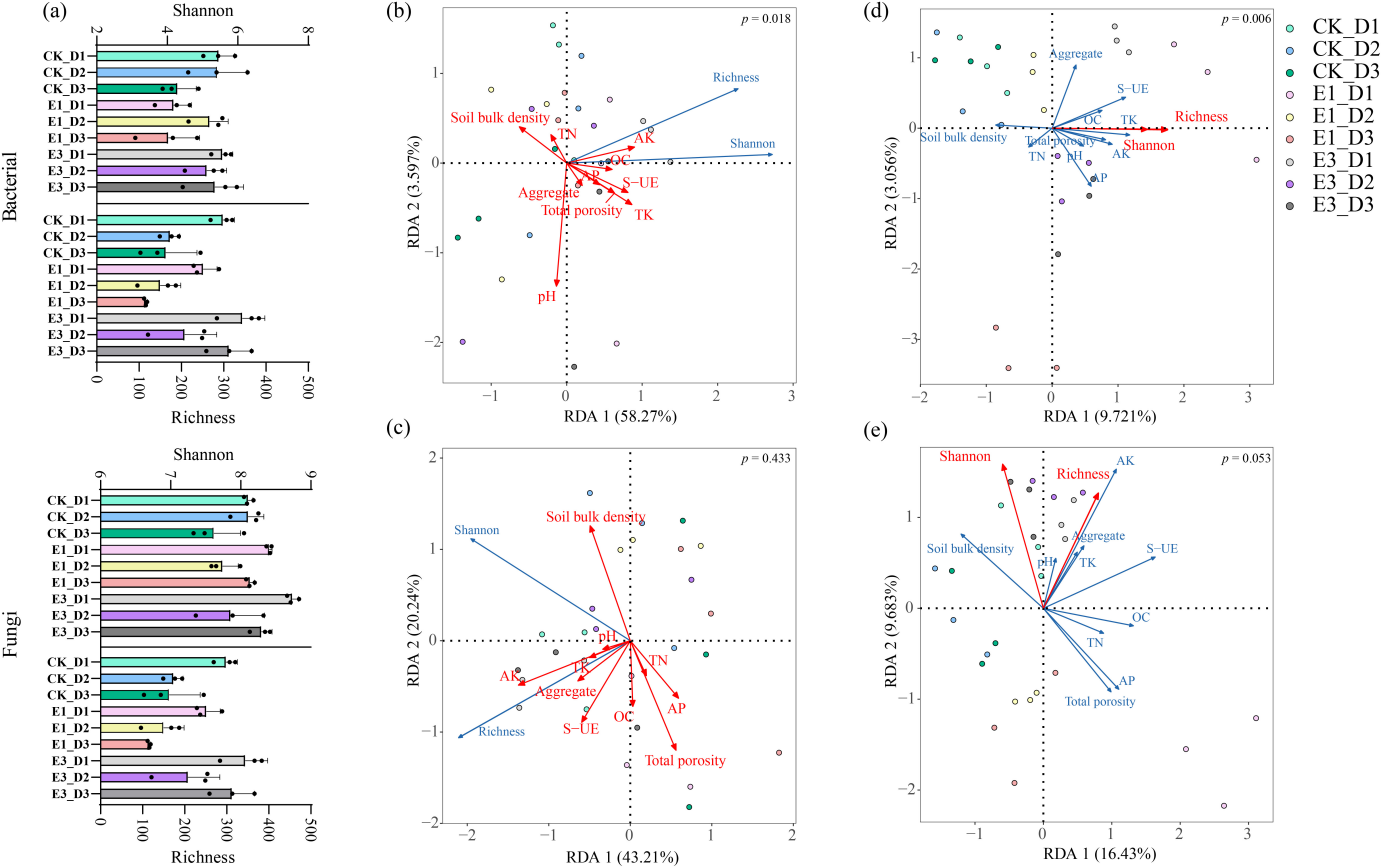
The α diversity of soil bacterial and fungal communities under different plantation ages, as well as the effects of soil physical and chemical factors on the α diversity obtained through multiple regression analysis. **(a)** Shannon index of microbial communities under different stand ages and soil depths. **(b)** Redundancy analysis (RDA) of soil chemical properties against alpha diversity indices for bacterial communities. **(c)** Redundancy analysis (RDA) of soil chemical properties against alpha diversity indices for fungal communities. **(d)** Redundancy analysis (RDA) of alpha diversity indices against soil chemical properties for bacterial communities. **(e)** Redundancy analysis (RDA) of alpha diversity indices against soil chemical properties for fungal communities.

For bacterial communities, significant differences in richness and Shannon index were observed among plots (Figure 3, Supplementary Table 3). Bacterial richness was highest in E3_D1 (859.33 ± 39.95), which was comparable to that in E1_D1 (794.33 ± 71.84) and E3_D3 (779.67 ± 16.07), whereas values in CK_D3 (564.00 ± 82.21) and E1_D2 (577.00 ± 64.37) were significantly lower (*p* < 0.05). The bacterial Shannon index was likewise highest in E3_D1 (8.74 ± 0.09) and lowest in CK_D3 (7.62 ± 0.38). For fungal communities, richness also differed significantly among plots, with E3_D1 exhibiting the highest value (344.33 ± 52.94) and E1_D3 the lowest (115.67 ± 3.51). In contrast, no significant differences in fungal Shannon index were detected among the plots.

To investigate the relationships between soil physicochemical properties and microbial alpha diversity, redundancy analyses were performed (Figure 3, Supplementary Table 4). The results showed that the explanatory model of soil physicochemical properties for bacterial alpha diversity was significant (*p* = 0.018), the first two axes of RDA accounted for 58.27% and 3.60% respectively of the variation in bacterial α-diversity, with a cumulative explained variance of 61.87%, indicating that the selected soil physicochemical factors collectively exerted a significant effect on bacterial diversity. Specifically, TK, AP, AK, OC, S-UE, total porosity, and aggregates were positively correlated with the first axis, whereas TN and bulk density were negatively correlated with the first axis. However, the correlations between bacterial diversity indices and soil physicochemical factors were not significant (*p* > 0.05). Soil physicochemical properties also produced a significant explanatory model for fungal alpha diversity (*p* = 0.006), the first two axes of RDA explained 9.72% and 3.06% of the variation in fungal alpha diversity respectively, with a cumulative explained variance rate of 12.78%, in which AK, bulk density, and total porosity exerted significant effects on fungal alpha diversity, indicating that these factors were important drivers of fungal diversity variation. Similarly, the correlations between fungal diversity indices and soil physicochemical factors were not significant (*p* > 0.05). At the same time, we conducted a multivariate linear regression analysis, and the results showed that TN was a positive effect factor common to both bacterial and fungal communities (bacterial Richness: β = 0.412, Shannon: β = 0.487; fungal Richness: β = 0.438, Shannon: β = 0.512), while aggregate content and bulk density were the main common negative effect factors. The S-UE had a stronger positive effect on the Shannon index (bacterial β = 0.378; fungal β = 0.401) than on Richness; pH only had a negative effect on fungal Richness (β = −0.156), but had no significant effect on bacteria; total porosity only had a weak positive effect on fungal Richness (β = 0.121), indicating that the combined effect of soil physical and chemical factors rather than the independent effect of a single factor was the key to regulating microbial alpha diversity, and the response of fungal diversity to soil structure factors (pH, porosity) was more sensitive than that of bacteria (Supplementary Table 4).

### 3.4 Screening of dominant microbial communities and core genera

Bacterial communities: At the phylum level, the dominant bacterial phyla were mainly Chloroflexota and Pseudomonadota. The relative abundance of Chloroflexota in E1_D1 (0.171 ± 0.040) and E3_D1 (0.181 ± 0.035) was significantly lower than that in CK_D1 (0.288 ± 0.077) (*p* < 0.05), whereas the relative abundance of Pseudomonadota in E1_D1 (0.353 ± 0.072) was significantly higher than that in CK_D2 (0.178 ± 0.017) and CK_D3 (0.162 ± 0.071) (*p* < 0.05). At the genus level, Candidatus Udaeobacter, Pedomicrobium, and Candidatus Xiphinematobacter were identified as dominant genera. The relative abundance of Candidatus Udaeobacter in E3_D1 (0.134 ± 0.038) was significantly higher than that in CK_D1 (0.055 ± 0.019) and the other treatments (*p* < 0.05). Pedomicrobium was significantly enriched in E1_D1 (0.078 ± 0.012) and E1_D2 (0.080 ± 0.008) (*p* < 0.05), whereas the relative abundance of Candidatus Xiphinematobacter in E1_D2 (0.009 ± 0.001) was significantly lower than that in CK_D2 (0.091 ± 0.012) (*p* < 0.05) (Supplementary Table 5).

Fungal communities: At the phylum level, the dominant fungal phyla included *Fungi_phy_Incertae_sedis* and *Mucoromycota*. The relative abundance of *Fungi_phy_Incertae_sedis* in E1_D1 (0.580 ± 0.136) was significantly higher than that in all CK treatments (0.039–0.139) (*p* < 0.05), whereas *Mucoromycota* was significantly reduced across all *E. grandis × urophylla* plantation plots (0.001–0.005) and remained lower than that observed in CK treatments (0.044–0.061) (*p* < 0.05). At the genus level, the dominant genera included *Fungi_gen_Incertae_sedis*, *Saitozyma*, *Laccaria*, *Melanconiella*, and *Mortierella*. *Fungi_gen_Incertae_sedis* was significantly enriched in E1_D1 (0.687 ± 0.127) (*p* < 0.05). The relative abundance of *Saitozyma* in CK_D1 (0.222 ± 0.107) was significantly higher than that in most *E. grandis × urophylla* plantation plots (*p* < 0.05). *Laccaria* was detected only in the *E. grandis × urophylla* plantation plots, with the highest relative abundance recorded in E1_D2 (0.229 ± 0.036). The relative abundance of *Melanconiella* in E1_D2 (0.129 ± 0.080) was significantly higher than that in the CK treatments (*p* < 0.05), and *Mortierella* was significantly enriched in E1_D2 (0.115 ± 0.048) (*p* < 0.05).

**FIGURE 4:**
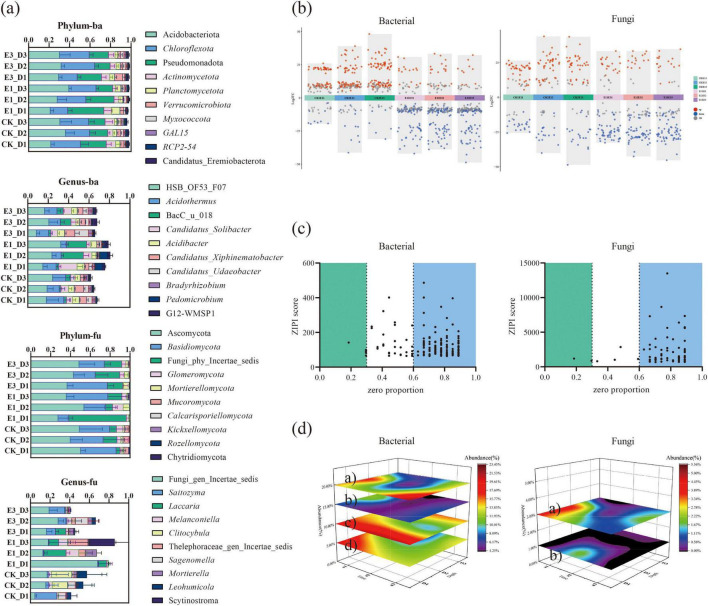
Abundance of dominant soil microbial communities and screening of core genera under different plantation ages. **(a)** Relative abundances of dominant phyla and genera of microbial communities across different stand ages and soil depths. **(b)** ASVs with significant intergroup differences screened via DESeq2. **(c)** Keystone microorganisms screened using the Zi-Pi method. **(d)** Relative abundance distribution of core microorganisms along the gradients of stand age and soil depth. Bacterial core genera: a) *Candidatus_Xiphinematobacter*, b) *Granulicella*, c) *Acidibacter*, d) *Acidothermus*; Fungal core genera: a) *Rozellomycota*, b) *Glutinomyces*.

As shown in Figure 4, core amplicon sequence variants (ASVs) were screened by comparing CK with E1 and E1 with E3 at the same soil depth, using thresholds of fold change >2 and *p* < 0.01. The results showed that among the differentially abundant ASVs identified between CK and E1, significant upregulation of relative abundance was the predominant pattern, whereas among the differentially abundant ASVs identified between E1 and E3, significant downregulation became the dominant trend. Among them, 9 bacterial ASVs (ASV936, ASV976, ASV526, ASV669, ASV129, ASV171, ASV1985, ASV890, ASV893) and 11 fungal ASVs (ASV3, ASV118, ASV7, ASV63, ASV1022, ASV290, ASV1069, ASV1066, ASV2193, ASV2763, ASV45) were retained as core microbial ASVs. Correcting statistical screening logic errors: Based on the analysis results of ZIPI, the bacterial core ASVs (ASV936, ASV976, ASV669, ASV171) and the fungal core ASV (ASV7, ASV290) were finally selected. A total of four core bacterial genera (*Acidothermus*, *Acidibacter*, *Candidatus Xiphinematobacter*, and *Granulicella*) and two core fungal genera (*Rozellomycota* and *Glutinomyces*) were identified, all of which, except for *Candidatus Xiphinematobacter*, were classified as rare taxa.

The relative abundance of Acidothermus in the CK_D1 soil layer was 17.20%, decreased to 11.96% in E3_D1, and then increased to 12.16% in E3_D3, indicating an initial decline followed by partial recovery with increasing plantation age, along with a downward shift in distribution toward deeper soil layers. Acidibacter showed relatively high abundance across all CK soil layers (2.00%–8.90%), with its relative abundance declining as plantation age increased. A comparatively high abundance was maintained only in E3_D1 (6.69%), indicating enrichment in the surface layer. The relative abundance of Candidatus Xiphinematobacter reached 10.40% in CK_D2, declined markedly in E1, and recovered to 10.05% in E3_D1. Granulicella showed generally low overall abundance but exhibited slight accumulation in E1_D2 and E3_D3, with an increasing tendency to shift toward the middle and deep soil layers as plantation age increased. Among the fungal genera, Rozellomycota showed relatively high abundance in CK (0.09%–0.47%), and its abundance declined significantly with increasing plantation age, indicating strong sensitivity to plantation age. Glutinomyces was detected only in E1_D1 (5.60%) and was absent from all other soil layers in the remaining sampling periods, indicating specific responses to both plantation age and soil depth.

### 3.5 Structural equation model fitting and functional interactions

Complex interactive relationships were observed among soil pH, potassium, and OC, with S-UE serving as a key mediator by significantly promoting the formation and stabilization of soil aggregates. The analytical results (Fisher’s *C* = 50.789, df = 46, *p* = 0.291; global χ^2^ = 34.187, df = 23, *p* = 0.062) indicated a good model fit and reasonably reflected the causal relationships among variables. Plantation age exerted significant positive effects on soil pH (Estimate = 0.0999, *p* < 0.01), TK (Estimate = 0.740, *p* < 0.001), and OC (Estimate = 2.537, *p* < 0.001), while exerting a negative effect on soil aggregates (Estimate = −2.682, *p* < 0.001). Soil depth had positive effects on pH (Estimate = 0.1222, *p* < 0.01) and TK (Estimate = 0.3822, *p* < 0.01), but a negative effect on S-UE (Estimate = −0.0273, *p* < 0.001). Along the intervariable pathways, both TK and pH exerted significant negative effects on OC (Estimate = −3.064 and −6.233, respectively; *p* < 0.01 or *p* < 0.001), whereas OC and pH exerted significant positive effects on S-UE (Estimate = 0.0139 and 0.0936, respectively; *p* < 0.001), and S-UE in turn exerted a highly significant positive effect on soil aggregates (Estimate = 179.289, *p* < 0.001).

**FIGURE 5:**
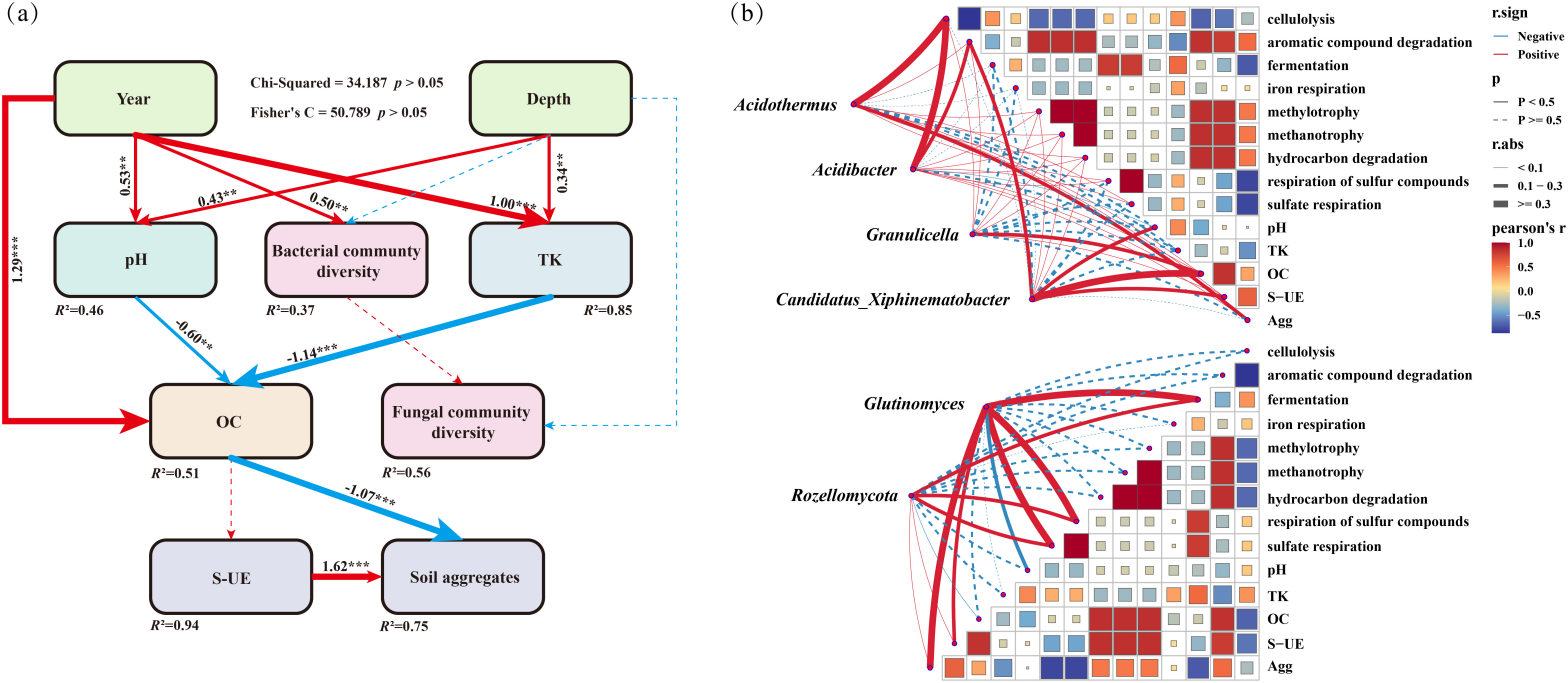
Structural model fitting under the combined influence of different plantation ages and soil depths (**a**), and a functional interaction network among core microbial genera, soil physicochemical properties (**b**). The structural equation model illustrated the effects of plantation age and soil depth on ecosystem multifunctionality (Fisher’s *C* = 50.789, *p* = 0.291, df = 46). Statistical significance was evaluated using a two-tailed Fisher’s *C*-test based on 46 independent soil samples. Red arrows indicate significant positive effects (*p* < 0.05), blue arrows indicate significant negative effects (*p* < 0.05), and dashed arrows indicate non-significant relationships. The numerical values adjacent to the arrows represent standardized path coefficients, and arrow widths are proportional to the magnitude of the path coefficients. *R*^2^ represents the proportion of explained variance. Significance levels for each predictor variable are indicated as follows: **p < 0.01, ***p < 0.001.

Distinct differentiation in functional adaptation and environmental responses was observed among different microbial taxa (Figure 5). At the functional level, Acidothermus showed a significant positive correlation with cellulose degradation (*r* = 0.456), whereas Acidibacter and Granulicella also showed positive correlations with this function, albeit with smaller magnitudes. Glutinomyces showed exceptionally strong positive correlations with fermentation (*r* = 0.779) and sulfur respiration (*r* = 1.000), and Rozellomycota likewise showed a positive correlation with sulfur respiration (*r* = 0.286), indicating its potential involvement in the sulfur cycle. In contrast, the functional associations of Candidatus Xiphinematobacter were relatively restricted, involving only aromatic compound degradation and methylotrophy. In terms of responses to environmental factors, Candidatus Xiphinematobacter showed a significant positive correlation with OC content (*r* = 0.360), Glutinomyces showed a strong positive correlation with soil aggregates (*r* = 0.802), whereas Granulicella showed a significant negative correlation with soil aggregates (*r* = −0.217), reflecting contrasting response patterns of different taxa to soil structure.

## 4 Discussion

### 4.1 Long-term cultivation of *E. grandis × urophylla* promotes TK accumulation and pH elevation in soil, while AP, OC, and enzyme activities exhibit dual attenuation with increasing plantation age and soil depth

Regarding plantation age, TK content showed a continuous increase as plantation age advanced and was significantly higher in the third-rotation *E. grandis × urophylla* plantation plots (E3) than in CK. A similar pattern of potassium accumulation under long-term cultivation was also reported by Kong et al. in Eucalyptus plantations, where TK content in third-rotation plantations exceeded that in first-rotation plantations, consistent with the findings of the present study. We hypothesize that this pattern may partially reflect the residual effects of compound fertilizers applied during the first and second rounds of afforestation, with potassium potentially entering the soil matrix through mineral fixation or profile migration. This residual effect, coupled with the inherent high spatial heterogeneity of soil mineral composition even under similar parent material conditions, has led to the increased TK levels observed in older forest plots. (Kong et al., 2022). Both AP and OC reached their highest values in the first-rotation plantation plots (E1) and then declined, showing a pattern of “initial improvement followed by subsequent degradation”. A similar trend was reported by Wang et al. in a study of soil nutrient dynamics in subtropical plantations, in which surface soil OC content in second-rotation Eucalyptus plantations decreased by approximately 12% relative to first-rotation plantations, whereas AP decreased by approximately 18%. That study suggested that, during the later stages of continuous cropping, the gradual decline in surface soil available nutrients and OC was associated with increased nutrient demand by trees, shifts in microbial community structure, and reduced organic matter decomposition rates (Wang et al., 2025). A meta-analysis by Xue et al., which integrated 2,744 observations from Eucalyptus plantation sites worldwide, showed that soil OC and TK were key factors regulating microbial community diversity (Xue et al., 2025). In this study, the significantly higher pH in E3 compared to CK and E1, while contradicting reports of widespread acidification from continuous Eucalyptus planting, may be due to the replacement of acidic litter from the previous stand with faster-decomposing, more cation-rich Eucalyptus litter. Eucalyptus leaves and litter then absorb Ca^2 +^, Mg^2 +^, and K^+^ from the subsoil via deep roots and return them to the topsoil through litter, driving surface alkalization. Increased S-UE activity in Eucalyptus forests may promote urea hydrolysis to produce NH_4_^+^, a process that consumes H^+^ and locally increases soil pH. It is important to emphasize that this pH increase is limited to the surface layer and does not reflect a reduction in soil acidification; deeper or rhizosphere acidification may be masked by depth-averaged sampling (Sauvadet et al., 2025). The CK stand originated from natural regeneration after logging and has remained undisturbed since 1979, whereas the E1 and E3 stands were established after clear-cutting, site preparation, tillage, and replanting. These pre-planting disturbances may have altered soil physicochemical baselines through mechanisms such as mixing of organic horizons with mineral soil, changes in litter quality and quantity, and initial fertilization effects, all of which could influence subsequent pH trajectories.

Regarding soil depth, TK content increased with soil depth, whereas OC and AP decreased significantly as soil depth increased. This vertical distribution pattern was consistent with the findings of Chen et al. in a study of soil profiles in plantations in the red soil region of Fujian Province (Chen and Yang, 2013), in which surface OC content was approximately 1.3-fold that of the deeper layers and surface AP content was approximately 1.2-fold that of the deeper layers, indicating that surface soils were enriched in nutrients through litter return and fertilizer input, whereas deeper soils were mainly regulated by parent material and leaching processes. A study by Ma et al. on rhizosphere and non-rhizosphere soils in young *Eucalyptus* plantations showed that OC and TN contents were significantly higher in the surface layer than in the middle layer, with differences of approximately 25%–30%, and that nutrient contents in non-rhizosphere soils were generally higher than those in rhizosphere soils, indicating that soil depth is an important factor affecting the physicochemical properties and bacterial abundance of *Eucalyptus* plantation soils (Ma et al., 2022). S-UE, S-ACP, and S-SC activities decreased significantly with increasing soil depth, consistent with the findings of Liu et al., in which S-SC, S-ACP, and S-UE activities in surface soils (0–20 cm) were all higher than those in middle-layer soils (20–40 cm), and enzyme activities in mixed forests were significantly higher than those in *Eucalyptus* monocultures (Liu et al., 2024). The marked decline in S-UE, S-ACP, and S-SC activities with increasing soil depth was also consistent with the findings of Cheng et al. on soil enzyme activities under different management regimes in *Eucalyptus* plantations, in which OC, TN, AP contents, and S-SC activity all decreased significantly after multiple consecutive rotations of *Eucalyptus* monoculture. Compared with monoculture, mixed planting of *Eucalyptus* with native broadleaf tree species significantly enhanced soil enzyme activities, indicating that mixed forests may help alleviate soil quality degradation caused by long-term monoculture cultivation (Cheng et al., 2023).

### 4.2 Long-term *E. grandis × urophylla* plantation restores soil bacterial diversity to relatively high levels, with a driving relationship between microbial communities and soil physicochemical properties

Both the richness and Shannon index of the soil bacterial community were highest in the surface layer of the third-rotation plantation plots (E3_D1) and lowest in the deep layer of the CK plots. This pattern partially differed from the findings of Xu et al. on soil microbial communities under multiple rotations of *Eucalyptus* plantations, in which successive rotations were reported to cause significant declines in soil multifunctionality as well as in the diversity and richness of fungal communities, while microbial network complexity, robustness, and intertaxon associations were markedly reduced in the third- and fourth-rotation stands. Notably, as the rotation number increased, soil bacterial communities shifted from carbon-utilization-dominated to nitrogen-utilization-dominated types, whereas fungal communities shifted from saprotrophic and pathogenic guilds toward symbiotic guilds (Xu et al., 2022). In this study, microbial diversity in the third-rotation plots (E3) was higher than that in the first-rotation plots, a trend broadly consistent with the recovery trajectory reported by Xu et al. Additionally, Xu et al. reported that the alpha diversity of fungal communities was more sensitive to soil environmental conditions than that of bacterial communities, with OC and TK identified as the main factors affecting fungal diversity (Xu et al., 2022).

In terms of the relationships between soil physicochemical properties and microbial community alpha diversity, the results of the present study consistently showed that soil physicochemical properties exerted significant effects on microbial community alpha diversity, whereas no significant correlations were detected between microbial alpha diversity and soil physicochemical properties. It was important to note that these findings are based on correlative analyses conducted within an observational study framework; therefore, they did not establish causal relationships. In a study of soil profiles in subalpine meadows, in which soil physicochemical properties, particularly pH and soil moisture content, were identified as the primary determinants of microbial community structure, explaining 47.24% to 63.75% of the variance, while the feedback effects of microbial diversity on soil properties did not reach statistical significance (Liang et al., 2026). Similarly, Yu et al. confirmed in a study of soil microbial communities that bacterial and fungal communities were primarily driven by abiotic factors, including altitude, pH, ammonium nitrogen, and TP, rather than by reverse microbial regulation of soil properties (Yu et al., 2026). Additionally, a cross-domain study by Tortosa et al. on soil biodiversity across six contrasting ecosystems showed that the co-variation among biological groups such as bacteria and fungi was limited at the alpha diversity level. However, beta diversity exhibited significant spatial coupling, which was primarily governed by differences in soil moisture conditions rather than by active biotic modification of the environment (Tortosa et al., 2026).

### 4.3 Compositional succession, genus-level migration, and functional decline of microbial communities under long-term *E. grandis × urophylla* plantation

The succession of microbial communities under long-term *E. grandis × urophylla* plantations indicated a multidimensional trend of soil degradation. At the bacterial phylum level, the relative abundance of oligotrophic *Chloroflexota* was significantly reduced in *Eucalyptus* plots, whereas copiotrophic *Pseudomonadota* was significantly enriched in the first-rotation plantation plots. This pattern is generally associated with rapid mineralization of soil organic matter and unusually high carbon and nitrogen turnover rates, which may represent an early signal of soil functional degradation. Within the fungal community, saprotrophic *Mucoromycota* declined significantly across all *Eucalyptus* plots. As the plantation rotation number increased, S-SC activity decreased significantly (by 14.00% to 64.29%), and the soil multifunctionality index declined by 0.61 (*p* < 0.01), indicating that long-term *E. grandis × urophylla* cultivation exerts multidimensional inhibitory effects on soil functionality.

Regarding the response patterns of core genera, *Acidothermus* was enriched in the surface layer of the control plots but showed reduced relative abundance in the first-rotation plantation plots (E1), before recovering and shifting to deeper soil layers in third-rotation plantations (E3). Similarly, *Candidatus Xiphinematobacter* was suppressed during the early stage of plantation establishment but recovered to levels approaching those of the control plots as plantation age increased. Analysis of changes in vertical distribution showed that the downward migration and loss of surface enrichment of these genera were often accompanied by impaired surface soil microhabitat function in the surface soil layer. Additionally, the abundance of *Rozellomycota* declined continuously with increasing years of monoculture, exhibiting an irreversible deterioration trend consistent with the sustained decline in soil nutrients caused by *Eucalyptus* monoculture (Wu et al., 2022). In terms of vertical distribution, *Acidothermus* and *Granulicella* shifted toward the middle and deeper soil layers as the years of monoculture increased, whereas *Acidibacter* remained enriched exclusively in the surface layer. Wang et al. showed that organic matter input and soil aggregate composition strongly influence the distribution patterns of microbial communities and that surface soil microbial communities are the most sensitive to changes in vegetation type. When the structure of the surface microbial community is disrupted, certain genera with strong adaptability or specific niche requirements shift to deeper layers to avoid unfavorable conditions, which provides further indirect evidence of degraded surface soil habitat quality. Different afforestation patterns significantly affect rhizosphere soil bacterial community structure, and factors such as total soil carbon, NO^–^_3_-N, AP, and pH can effectively explain variations in bacterial communities (Wang W. et al., 2022).

### 4.4 Chain regulation of soil physicochemical properties and specific responses of microbial functional differentiation under long-term *E. grandis* × urophylla plantation

Plantation age exerted significant positive effects on soil pH, TK, and OC, but a significant negative effect on aggregate stability. This finding differed from the conclusions of most studies on continuous *Eucalyptus* cropping. Zhang et al. (2024) examined soils under *E. grandis × urophylla* of different plantation age and reported that soil pH, organic matter, and AP content declined significantly with increasing plantation ages, whereas S-UE activity showed no significant difference. The complex root systems and long-term litter inputs in natural forests may have established a more stable organic matter pool. Therefore, even when continuous cropping caused structural degradation after the replacement of natural forests by *E. grandis × urophylla*, the absolute OC content may still have exceeded that of severely degraded natural forests (Xu et al., 2025). Additionally, the increase in pH with successive cropping cycles observed in this study contrasted with the findings of most previous studies, which may be attributed to the uptake and retranslocation of specific base cations by *Eucalyptus*, or to the acidic soil conditions inherited from the preceding mixed forests of *C. lanceolata* and *P. massoniana*, under which base cations introduced through litter decomposition after *Eucalyptus* establishment gradually increased pH from a relatively low baseline (Dai et al., 2023). Notably, TK and pH exerted negative effects on OC, whereas OC and pH positively influenced aggregate stability via regulating S-UE activity, reflecting the complexity of soil functional regulation under continuous cropping. Wang et al. reported that long-term *Eucalyptus* cultivation increased soil bulk density, decreased organic matter content and iron-aluminum-manganese oxide concentrations, and significantly reduced aggregate stability with successive rotation cycles. That study also identified a significant positive correlation between aggregate stability and organic matter content (Wang J. et al., 2022), consistent with the positive driving role of S-UE in aggregate stability observed in this study.

With respect to microbial functional differentiation, *Glutinomyces* was strongly and positively correlated with sulfur respiration and aggregate stability, whereas *Granulicella* was negatively correlated with aggregate stability. This differentiation may reflect a shift in microbial strategy from copiotrophy to oligotrophy under prolonged continuous cropping. Studies on microbial communities in continuously planted *Eucalyptus* stands have shown that soil microbial diversity decreases as rotation number increases, with rare bacterial diversity declining, whereas rare fungal diversity shows the opposite trend (Wang et al., 2024). The enrichment of *Glutinomyces*, as a fungal taxon, in the later stages of continuous cropping may be attributed to its capacity to utilize organic matter accumulated under *Eucalyptus* monoculture or to its involvement in sulfur transformation as an adaptation to nutrient stress (Jiang et al., 2024). In contrast, the decline of *Granulicella* was consistent with the greater ecological activity of this genus in natural forests, and its negative correlation with aggregate stability may reflect its sensitivity to disturbances associated with continuous cropping.

In response to the degradation associated with continuous *E. grandis × urophylla* plantation, including TK accumulation accompanied by AP decline, OC depletion, reduced enzyme activity, and microbial functional differentiation, effective soil remediation measures are essential for the sustainable management of *Eucalyptus* plantations. To address the degradation of microbial community structure caused by continuous *Eucalyptus* cropping, including the decline of copiotrophic *Pseudomonadota*, the reduction of saprotrophic *Mucoromycota*, and the downward shift of certain functional microbial genera, another effective approach is the targeted screening and inoculation of beneficial microorganisms with phosphate-solubilizing, potassium-solubilizing, nitrogen-fixing, or soilborne pathogen-antagonizing functions (Li et al., 2026).

This study has several limitations. only one plot was set up for each treatment, and the three sampled trees within each plot were actually sub-samples rather than independent biological replicates. The differences between treatments may partly have resulted from the heterogeneity of the microenvironment at the plot scale. Statistical inference was only applicable to the current stand. The control forest (CK) originated from the natural succession after historical logging and did not represent the undisturbed primary forest baseline. Before the establishment of *Eucalyptus* forests, a clear-cutting, land preparation, and fertilization were carried out. Therefore, the differences between CK and *Eucalyptus* forests may reflect the combined effect of clear-cutting interference, soil cultivation, and early fertilization, rather than rather than solely stand age or tree species conversion. In the future, additional plots for natural succession after clear-cutting and adjacent control plots from the original forest should be established, and combined with long-term monitoring to distinguish the individual effects of tree species conversion and forest management interference. The FAPROTAX functional prediction was based on cultivated reference organisms and failed to cover soil groups that are not cultivated or poorly characterized. Therefore, the functional inference in this study should be regarded as exploratory and qualitative description. Subsequent verification can be conducted by combining metagenomic or multi-omics methods.

## 5 Conclusion

Long-term *E. grandis × urophylla* monoculture induces systematic, multidimensional soil functional degradation. Soil TK accumulates continuously, whereas AP and OC exhibit a trend of “short-term improvement followed by long-term decline”. OC, AP, and the activities of S-UE, S-ACP, and S-SC all decrease with increasing soil depth. Although soil bacterial diversity is partially restored in long-term plantations, microbial communities undergo adaptive structural succession. Specifically, the relative abundances of copiotrophic Pseudomonadota, oligotrophic Chloroflexota, and saprotrophic Mucoromycota decrease, core functional genera migrate toward deeper soil layers, and surface soil microbial ecological functions are progressively weakened. Soil pH, TK, and OC serve as key regulatory factors shaping soil microbial communities. The co-occurrence of TK accumulation and pH elevation jointly inhibits OC accumulation. These cascading regulatory effects ultimately drive progressive degradative succession of the soil ecosystem under continuous Eucalyptus cultivation.

## Data Availability

The datasets presented in this study can be found in online repositories. The names of the repository/repositories and accession number(s) can be found in the article/Supplementary material.
