# Supplementary material for: Vertical differentiation of soil properties and microbial communities in long-term Eucalyptus grandis × urophylla plantations: surface diversity recovery does not offset deep-layer functional degradation
Source: Front Microbiol. 2026 Aug 5;17:1896384. doi: 10.3389/fmicb.2026.1896384 (PMC13485938; doi:10.3389/fmicb.2026.1896384)
Supplement: Supplementary file 1 [file Table_1.docx]

Table S1 Sample plot overview

| Code | Tree species | The original  tree species | Latitude and Longitude | | Slope  (°) | Aspect | Altitude  (m) | Diameter at  breast height  (cm) | Tree height  (m) | Crown width  （E-W）/m | Crown width（N-S）/m | Canopy coverage |
| --- | --- | --- | --- | --- | --- | --- | --- | --- | --- | --- | --- | --- |
|  |  |  | Longitude/E | Latitude/N |  |  |  |  |  |  |  |  |
| CK | *Cunninghamia lanceolata*  *Pinus massoniana* | - | 117.45731 | 24.34525662 | 12~25 | S171° | 565.4 | 16.10 | 12.25 | 2.98 | 2.80 | 0.76 |
| E1 | *Eucalyptus grandis* x *urophylla* | *Cunninghamia lanceolata*  *Pinus massoniana* | 117.44641487 | 24.34817664 | 10~27 | S168° | 478.9 | 11.52 | 13.50 | 2.16 | 2.48 | 0.71 |
| E3 | *Eucalyptus grandis* x *urophylla* | *Cunninghamia lanceolata*  *Pinus massoniana* | 117.44072325 | 24.33865813 | 24~37 | S197° | 340.5 | 10.51 | 12.62 | 2.05 | 2.01 | 0.65 |

Table S2-1 The soil physical and chemical properties of different planting plots

| Code | TN（g/kg） | TK（g/kg） | AP（mg/kg） | AK（mg/kg） | OC（g/kg） |
| --- | --- | --- | --- | --- | --- |
| CK_D1 | 1.26±0.08Aa | 0.74±0.13Ca | 31.27±8.33Ba | 0.13±0.00Ba | 46.51±0.40Cb |
| CK_D2 | 0.79±0.02Ab | 0.93±0.07Ca | 35.77±5.99Ba | 0.13±0.00Ba | 45.78±0.16Cb |
| CK_D3 | 0.96±0.16Aa | 0.41±0.05Cb | 33.44±1.06Ba | 0.14±0.00Ba | 48.26±0.95Ca |
| E1_D1 | 1.15±0.07Aa | 2.24±0.27Ba | 46.25±5.31Ab | 0.13±0.00Ba | 47.74±0.23Ac |
| E1_D2 | 0.79±0.15Ab | 0.97±0.14Bb | 33.18±5.06Ab | 0.13±0.00Ba | 50.24±0.67Ab |
| E1_D3 | 1.20±0.03Aa | 0.71±0.10Bb | 66.07±4.59Aa | 0.13±0.00Ba | 54.10±0.37Aa |
| E3_D1 | 0.85±0.07Ab | 2.48±0.29Ab | 32.77±7.46Ba | 0.14±0.00Aa | 46.60±0.27Bb |
| E3_D2 | 1.36±0.21Aa | 2.52±0.35Ab | 33.97±3.27Ba | 0.14±0.00Aa | 46.94±0.60Bb |
| E3_D3 | 0.91±0.03Aa | 2.80±0.29Aa | 28.29±5.84Ba | 0.14±0.00Aa | 48.94±0.77Ba |

Different capital letters indicate significant differences among the planting years at the same soil depth, while different lowercase letters indicate significant differences among the soil depths at the same planting year. (*p* < 0.05)

Table S2-2 The soil physical and chemical properties of different planting plots

| Code | pH | S-UE（U/g） | S-ACP（U/g） | S-SC（U/g） | SBD（g/cm^3^) | Tp（%） | Aggregate（%） |
| --- | --- | --- | --- | --- | --- | --- | --- |
| CK_D1 | 4.48±0.03Ba | 0.09±0.00Bb | 0.33±0.03Aa | 1.79±0.41Bc | 1.4±0.08Aab | 45.04±3.27Ba | 70.78±1.52Aa |
| CK_D2 | 4.52±0.04Ba | 0.08±0.01Bb | 0.30±0.01Aa | 2.93±0.31Bb | 1.43±0.05Aa | 44.63±1.47Ba | 69.42±1.72Aa |
| CK_D3 | 4.21±0.06Bb | 0.12±0.01Ba | 0.31±0.03Aa | 5.21±0.29Ba | 1.44±0.04Aa | 43.44±1.71Ba | 69.91±1.11Aa |
| E1_D1 | 4.26±0.07Ca | 0.07±0.01Ac | 0.17±0.01Ac | 7.18±0.46Ac | 1.35±0.02Ba | 46.98±0.72Aa | 55.82±1.27Cc |
| E1_D2 | 4.28±0.13Ca | 0.12±0.01Ab | 0.3±0.06Ab | 9.27±0.72Ab | 1.33±0.01Bab | 46.99±0.43Aa | 64.30±1.7Cb |
| E1_D3 | 4.40±0.09Ca | 0.23±0.01Aa | 0.47±0.01Aa | 12.53±0.3Aa | 1.23±0.04Bb | 50.49±1.29Aa | 73.85±1.79Ca |
| E3_D1 | 4.92±0.08Aa | 0.11±0.01Ab | 0.28±0.02Ac | 1.47±0.16Cb | 1.39±0.06Aab | 45.24±1.86Ba | 63.81±1.75Bb |
| E3_D2 | 4.76±0.02Aa | 0.14±0.01Aa | 0.30±0.06Ab | 3.17±0.31Ca | 1.42±0.02Aa | 44.46±0.53Ba | 67.02±0.82Ba |
| E3_D3 | 4.32±0.06Ab | 0.15±0.01Aa | 0.37±0.05Aa | 3.01±0.23Ca | 1.36±0.02Ab | 46.53±0.77Ba | 68.91±1.53Ba |

Different capital letters indicate significant differences among the planting years at the same soil depth, while different lowercase letters indicate significant differences among the soil depths at the same planting year. (*p* < 0.05)

Table S3 The alpha diversity of soil microorganisms in different planting plots

| Code | Bacterial | | Fungi | |
| --- | --- | --- | --- | --- |
|  | Richness | Shannon | Richness | Shannon |
| CK_D1 | 639.00±55.56c | 8.11±0.07bcd | 298.67±26.5ab | 5.46±0.45a |
| CK_D2 | 666.00±21.66bc | 8.11±0.22bcd | 173.33±22.72cd | 5.42±0.84a |
| CK_D3 | 564.00±82.21c | 7.62±0.38d | 163.67±73.22cd | 4.29±0.53a |
| E1_D1 | 794.33±71.84ab | 8.41±0.04ab | 251.67±32.58abc | 4.18±0.50a |
| E1_D2 | 577.00±64.37c | 7.74±0.22cd | 150.33±48.00cd | 5.20±0.53a |
| E1_D3 | 673.67±39.26bc | 8.13±0.06bcd | 115.67±3.51d | 4.03±0.88a |
| E3_D1 | 859.33±39.95a | 8.74±0.09a | 344.33±52.94a | 5.57±0.29a |
| E3_D2 | 625.00±89.20c | 7.86±0.48bcd | 208.00±75.39bcd | 5.12±0.56a |
| E3_D3 | 779.67±16.07ab | 8.30±0.15abc | 312.67±53.50ab | 5.35±0.81a |

Different lowercase letters indicate significant differences between groups, with *p* < 0.05.

Table S4-1 Bacterial community effects on soil physical and chemical properties and alpha redundancy analysis

| Indicators | RDA1 | RDA2 | r2 | pvals |  | Indicators | RDA1 | RDA2 | r2 | pvals |
| --- | --- | --- | --- | --- | --- | --- | --- | --- | --- | --- |
| TN | -0.795001453 | 0.606607525 | 0.0254 | 0.75 |  | Richness | 0.99996 | 0.00897322 | 0.3974 | 0.003 |
| TK | 0.924455932 | -0.381288906 | 0.2123 | 0.053 |  | Shannon | 0.999978 | 0.006609889 | 0.2711 | 0.022 |
| AP | 0.922163246 | -0.386800916 | 0.0548 | 0.507 |  |  |  |  |  |  |
| AK | 0.997536179 | -0.070153917 | 0.1642 | 0.113 |  |  |  |  |  |  |
| OC | 0.975044492 | -0.222009547 | 0.0809 | 0.353 |  |  |  |  |  |  |
| pH | 0.141876437 | -0.989884375 | 0.1994 | 0.075 |  |  |  |  |  |  |
| S-UE | 0.941869565 | -0.335978753 | 0.1721 | 0.086 |  |  |  |  |  |  |
| SBD | -0.909548563 | 0.415597657 | 0.1211 | 0.19 |  |  |  |  |  |  |
| Tp | 0.925578264 | -0.378556307 | 0.1134 | 0.217 |  |  |  |  |  |  |
| Aggregate | 0.831973701 | -0.554815069 | 0.0197 | 0.79 |  |  |  |  |  |  |
|  |  |  |  |  |  |  |  |  |  |  |
|  | Df | Inertia | F | Pr(>F) |  |  | Df | Inertia | F | Pr(>F) |
| Model | 10 | 1.856367781 | 2.597153527 | 0.018 |  | Model | 4 | 1.570119406 | 1.024410326 | 0.433 |
| Residual | 16 | 1.143632219 |  |  |  | Residual | 22 | 8.429880594 |  |  |

Table S4-2 The influence of bacterial communities on soil physical and chemical properties and the multivariate linear regression analysis of α diversity

| Richness | Coefficient (B) | Standard Error | t-value | p-value | VIF |  | Shannon | Coefficient (B) | Standard Error | t-value | p-value | VIF |
| --- | --- | --- | --- | --- | --- | --- | --- | --- | --- | --- | --- | --- |
| (Intercept) | 1247.32 | 985.64 | 1.265 | 0.234 | - |  | (Intercept) | 5.234 | 3.876 | 1.35 | 0.207 | - |
| TN | 156.2 | 68.41 | 2.284 | 0.041 | 2.87 |  | TN | 2.104 | 0.723 | 2.91 | 0.012 | 2.87 |
| TK | -18.94 | 22.17 | -0.854 | 0.413 | 1.56 |  | TK | -0.087 | 0.234 | -0.372 | 0.718 | 1.56 |
| AP | 0.87 | 1.23 | 0.707 | 0.496 | 1.78 |  | AP | 0.012 | 0.013 | 0.923 | 0.378 | 1.78 |
| AK | 187.43 | 245.61 | 0.763 | 0.462 | 1.33 |  | AK | 1.876 | 2.345 | 0.8 | 0.442 | 1.33 |
| OC | -2.34 | 3.12 | -0.75 | 0.47 | 2.45 |  | OC | -0.023 | 0.029 | -0.793 | 0.447 | 2.45 |
| pH | -45.67 | 38.92 | -1.173 | 0.267 | 1.89 |  | pH | -0.134 | 0.367 | -0.365 | 0.723 | 1.89 |
| S-UE | 87.34 | 112.56 | 0.776 | 0.455 | 2.03 |  | S-UE | 1.876 | 0.789 | 2.378 | 0.031 | 2.03 |
| SBD | -89.23 | 78.45 | -1.137 | 0.282 | 1.92 |  | SBD | -1.543 | 0.723 | -2.134 | 0.045 | 1.92 |
| Tp | 5.67 | 4.98 | 1.139 | 0.281 | 2.56 |  | Tp | 0.045 | 0.047 | 0.957 | 0.361 | 2.56 |
| Aggregate | -11.54 | 4.89 | -2.36 | 0.038 | 1.44 |  | Aggregate | -0.034 | 0.046 | -0.739 | 0.477 | 1.44 |
|  |  |  |  |  |  |  |  |  |  |  |  |  |
| Richness | Beta |  |  |  |  |  | Shannon | Beta |  |  |  |  |
| TN | 0.412 |  |  |  |  |  | TN | 0.487 |  |  |  |  |
| Aggregate | -0.298 |  |  |  |  |  | S-UE | 0.378 |  |  |  |  |
| Soil bulk density | -0.165 |  |  |  |  |  | Soil bulk density | -0.287 |  |  |  |  |
| S-UE | 0.143 |  |  |  |  |  | Aggregate | -0.089 |  |  |  |  |

Table S4-3 Fungal community effects on soil physical and chemical properties and alpha redundancy analysis

| Indicators | RDA1 | RDA2 | r2 | pvals |  | Indicators | RDA1 | RDA2 | r2 | pvals |
| --- | --- | --- | --- | --- | --- | --- | --- | --- | --- | --- |
| TN | 0.546965882 | -0.837154898 | 0.0331 | 0.656 |  | Richness | 0.633259298 | 0.773939701 | 0.5032 | 0.001 |
| TK | -0.959302669 | -0.282379868 | 0.0606 | 0.481 |  | Shannon | -0.07010968 | 0.997539289 | 0.3853 | 0.006 |
| AP | 0.769969992 | -0.638080098 | 0.1425 | 0.175 |  |  |  |  |  |  |
| AK | -0.962107488 | -0.272670464 | 0.4519 | 0.003 |  |  |  |  |  |  |
| OC | 0.045605386 | -0.998959533 | 0.088 | 0.358 |  |  |  |  |  |  |
| pH | -0.975836346 | -0.218502691 | 0.0264 | 0.711 |  |  |  |  |  |  |
| S-UE | -0.651828186 | -0.758366677 | 0.2144 | 0.067 |  |  |  |  |  |  |
| SBD | -0.442738167 | 0.896650944 | 0.3192 | 0.006 |  |  |  |  |  |  |
| Tp | 0.509472697 | -0.860486822 | 0.3099 | 0.012 |  |  |  |  |  |  |
| Aggregate | -0.880291549 | -0.474433123 | 0.1241 | 0.218 |  |  |  |  |  |  |
|  |  |  |  |  |  |  |  |  |  |  |
|  | Df | Inertia | F | Pr(>F) |  |  | Df | Inertia | F | Pr(>F) |
| Model | 10 | 3.848459532 | 2.861919328 | 0.006 |  | Model | 4 | 3.170355883 | 1.547350457 | 0.053 |
| Residual | 16 | 2.151540468 |  |  |  | Residual | 22 | 6.829644117 |  |  |

Table S4-4 The influence of fungi communities on soil physical and chemical properties and the multivariate linear regression analysis of α diversity

| Richness | Coefficient (B) | Standard Error | t-value | p-value | VIF |  | Shannon | Coefficient (B) | Standard Error | t-value | p-value | VIF |
| --- | --- | --- | --- | --- | --- | --- | --- | --- | --- | --- | --- | --- |
| (Intercept) | 1287.56 | 876.23 | 1.469 | 0.173 | - |  | (Intercept) | 6.789 | 4.123 | 1.647 | 0.131 | - |
| TN | 185.34 | 72.15 | 2.569 | 0.028 | 2.91 |  | TN | 2.456 | 0.789 | 3.113 | 0.011 | 2.91 |
| TK | -24.56 | 23.78 | -1.033 | 0.326 | 1.54 |  | TK | -0.112 | 0.245 | -0.457 | 0.658 | 1.54 |
| AP | 1.23 | 1.31 | 0.939 | 0.37 | 1.76 |  | AP | 0.018 | 0.014 | 1.286 | 0.227 | 1.76 |
| AK | 245.78 | 258.34 | 0.951 | 0.364 | 1.31 |  | AK | 2.134 | 2.567 | 0.831 | 0.425 | 1.31 |
| OC | -3.12 | 3.34 | -0.934 | 0.373 | 2.43 |  | OC | -0.031 | 0.032 | -0.969 | 0.356 | 2.43 |
| pH | -58.34 | 41.23 | -1.415 | 0.188 | 1.87 |  | pH | -0.156 | 0.389 | -0.401 | 0.697 | 1.87 |
| S-UE | 112.45 | 118.67 | 0.948 | 0.365 | 2.01 |  | S-UE | 2.145 | 0.823 | 2.606 | 0.026 | 2.01 |
| Soil bulk density | -95.67 | 82.34 | -1.162 | 0.272 | 1.9 |  | Soil bulk density | -1.789 | 0.756 | -2.366 | 0.039 | 1.9 |
| Total porosity | 7.34 | 5.23 | 1.403 | 0.191 | 2.54 |  | Total porosity | 0.056 | 0.049 | 1.143 | 0.279 | 2.54 |
| Aggregate | -13.45 | 5.12 | -2.627 | 0.025 | 1.42 |  | Aggregate | -0.045 | 0.048 | -0.938 | 0.37 | 1.42 |
|  |  |  |  |  |  |  |  |  |  |  |  |  |
| Richness | Beta |  |  |  |  |  | Shannon | Beta |  |  |  |  |
| TN | 0.438 |  |  |  |  |  | TN | 0.512 |  |  |  |  |
| Aggregate | -0.312 |  |  |  |  |  | S-UE | 0.401 |  |  |  |  |
| Soil bulk density | -0.184 |  |  |  |  |  | Soil bulk density | -0.301 |  |  |  |  |
| pH | -0.156 |  |  |  |  |  | AK | 0.145 |  |  |  |  |
| S-UE | 0.148 |  |  |  |  |  | Aggregate | -0.098 |  |  |  |  |
| Total porosity | 0.121 |  |  |  |  |  |  |  |  |  |  |  |

Table S5-1 Distribution of dominant bacterial communities at the phylum and genus levels of the bacterial community

| Phylum | CK_D1 | CK_D2 | CK_D3 | E1_D1 | E1_D2 | E1_D3 | E3_D1 | E3_D2 | E3_D3 |
| --- | --- | --- | --- | --- | --- | --- | --- | --- | --- |
| Acidobacteriota | 0.22±0.03a | 0.36±0.08a | 0.31±0.11a | 0.21±0.03a | 0.28±0.09a | 0.40±0.02a | 0.30±0.03a | 0.32±0.03a | 0.30±0.10a |
| Chloroflexota | 0.29±0.08ab | 0.23±0.05ab | 0.28±0.05ab | 0.17±0.04b | 0.28±0.05ab | 0.26±0.02ab | 0.18±0.03b | 0.33±0.04a | 0.29±0.04ab |
| Proteobacteria | 0.25±0.05ab | 0.18±0.02b | 0.16±0.07b | 0.35±0.07a | 0.28±0.09ab | 0.16±0.04b | 0.24±0.04ab | 0.15±0.04b | 0.19±0.05b |
| Actinomycetota | 0.09±0.04a | 0.05±0.01a | 0.09±0.09a | 0.07±0.01a | 0.05±0.01a | 0.03±0.01a | 0.08±0.02a | 0.06±0.02a | 0.06±0.02a |
| Planctomycetota | 0.04±0.01ab | 0.04±0.01ab | 0.02±0.01b | 0.08±0.05a | 0.04±0.01ab | 0.05±0.01ab | 0.05±0.02ab | 0.04±0.01ab | 0.03±0.01b |
| Verrucomicrobiota | 0.04±0.01a | 0.04±0.01a | 0.01±0.01a | 0.04±0.02a | 0.01±0.00a | 0.01±0.01a | 0.08±0.03a | 0.03±0.01a | 0.03±0.01a |
| Myxococcota | 0.01±0.00a | 0.03±0.01a | 0.03±0.00a | 0.02±0.01a | 0.03±0.01a | 0.03±0.00a | 0.02±0.01a | 0.03±0.01a | 0.03±0.02a |
| GAL15 | 0.01±0.01bc | 0.02±0.01ab | 0.03±0.01a | 0.00±0.00c | 0.01±0.00bc | 0.02±0.01ab | 0.01±0.01bc | 0.02±0.00ab | 0.02±0.01ab |
| RCP2-54 | 0.01±0.01a | 0.02±0.01a | 0.01±0.01a | 0.01±0.01a | 0.01±0.01a | 0.01±0.00a | 0.01±0.00a | 0.01±0.00a | 0.01±0.00a |
| Candidatus_Eremiobacterota | 0.01±0.00ab | 0.01±0.00ab | 0.02±0.01a | 0.01±0.00ab | 0.00±0.00b | 0.01±0.00ab | 0.02±0.01a | 0.01±0.00ab | 0.01±0.01ab |
|  |  |  |  |  |  |  |  |  |  |
| Genus | CK_D1 | CK_D2 | CK_D3 | E1_D1 | E1_D2 | E1_D3 | E3_D1 | E3_D2 | E3_D3 |
| HSB_OF53_F07 | 0.18±0.12a | 0.19±0.06a | 0.25±0.2a | 0.15±0.04a | 0.24±0.04a | 0.33±0.05a | 0.09±0.03a | 0.21±0.08a | 0.17±0.04a |
| *Acidothermus* | 0.17±0.06a | 0.12±0.05ab | 0.13±0.04ab | 0.13±0.01ab | 0.09±0.02ab | 0.06±0.01ab | 0.12±0.03b | 0.11±0.04ab | 0.12±0.04ab |
| BacC_u_018 | 0.03±0.03bc | 0.02±0.01c | 0.05±0.04bc | 0.03±0.02bc | 0.22±0.05a | 0.20±0.02a | 0.02±0.01bc | 0.11±0.06b | 0.06±0.01bc |
| *Candidatus_Solibacter* | 0.06±0.03b | 0.08±0.03b | 0.04±0.02b | 0.18±0.05a | 0.08±0.04b | 0.04±0.00b | 0.07±0.01b | 0.05±0.02b | 0.08±0.02b |
| *Acidibacter* | 0.07±0.01a | 0.05±0.01a | 0.05±0.04a | 0.07±0.01a | 0.04±0.02a | 0.06±0.02a | 0.07±0.01a | 0.04±0.02a | 0.05±0.01a |
| *Candidatus_Xiphinematobacter* | 0.07±0.01ab | 0.09±0.01a | 0.03±0.02bc | 0.05±0.03abc | 0.01±0.00c | 0.02±0.00bc | 0.1±0.04a | 0.06±0.00abc | 0.06±0.03abc |
| *Candidatus_Udaeobacter* | 0.06±0.02b | 0.05±0.01b | 0.01±0.01b | 0.04±0.02b | 0.00±0.00b | 0.02±0.02b | 0.13±0.04a | 0.03±0.01b | 0.05±0.03b |
| *Bradyrhizobium* | 0.04±0.03a | 0.03±0.01a | 0.03±0.02a | 0.02±0.01a | 0.02±0.01a | 0.01±0.00a | 0.04±0.01a | 0.03±0.01a | 0.05±0.01a |
| *Pedomicrobium* | 0.01±0.00c | 0.01±0.00c | 0.01±0.00c | 0.08±0.01a | 0.08±0.01a | 0.03±0.01b | 0.01±0.00c | 0.00±0.00c | 0.01±0.00c |
| G12-WMSP1 | 0.00±0.00b | 0.01±0.01ab | 0.02±0.01ab | 0.02±0.01ab | 0.03±0.02ab | 0.03±0.02ab | 0.01±0.01ab | 0.05±0.02a | 0.02±0.01ab |

Different lowercase letters indicate significant differences between groups, with *p* < 0.05.

Table S5-2 Distribution of dominant bacterial communities at the phylum and genus levels of fungal communities

| Phylum | CK_D1 | CK_D2 | CK_D3 | E1_D1 | E1_D2 | E1_D3 | E3_D1 | E3_D2 | E3_D3 |
| --- | --- | --- | --- | --- | --- | --- | --- | --- | --- |
| Ascomycota | 0.5±0.05a | 0.40±0.12a | 0.49±0.23a | 0.28±0.10a | 0.54±0.21a | 0.36±0.08a | 0.37±0.06a | 0.43±0.11a | 0.49±0.16a |
| Basidiomycota | 0.36±0.07a | 0.33±0.19a | 0.31±0.35a | 0.11±0.04a | 0.22±0.10a | 0.42±0.18a | 0.4±0.06a | 0.22±0.13a | 0.25±0.06a |
| Fungi_phy_Incertae_sedis | 0.04±0.01b | 0.14±0.11b | 0.07±0.06b | 0.58±0.14a | 0.07±0.05b | 0.14±0.19b | 0.16±0.05b | 0.25±0.04b | 0.18±0.07b |
| Glomeromycota | 0.02±0.01a | 0.04±0.03a | 0.06±0.05a | 0.02±0.01a | 0.11±0.10a | 0.05±0.03a | 0.01±0.00a | 0.05±0.03a | 0.04±0.03a |
| Mortierellomycota | 0.02±0.01a | 0.02±0.01a | 0.02±0.01a | 0.01±0.00a | 0.06±0.01a | 0.01±0.00a | 0.06±0.05a | 0.04±0.02a | 0.02±0.00a |
| Mucoromycota | 0.05±0.02a | 0.06±0.03a | 0.04±0.02a | 0.00±0.00b | 0.00±0.00b | 0.00±0.00b | 0.00±0.00b | 0.00±0.00b | 0.00±0.00b |
| Calcarisporiellomycota | 0.00±0.00b | 0.00±0.00b | 0.01±0.01ab | 0.00±0.00b | 0.00±0.00b | 0.00±0.00b | 0.00±0.00b | 0.01±0.01ab | 0.01±0.00a |
| Kickxellomycota | 0.00±0.00a | 0.00±0.00a | 0.00±0.00a | 0.00±0.00a | 0.00±0.00a | 0.02±0.03a | 0.00±0.00a | 0.00±0.00a | 0.00±0.00a |
| Rozellomycota | 0.01±0.01a | 0.00±0.00b | 0.00±0.00b | 0.00±0.00b | 0.00±0.00b | 0.00±0.00b | 0.00±0.00b | 0.00±0.00b | 0.00±0.00b |
| Chytridiomycota | 0.00±0.00a | 0.00±0.00a | 0.00±0.00a | 0.00±0.00a | 0.00±0.00a | 0.00±0.00a | 0.00±0.00a | 0.00±0.00a | 0.00±0.00a |
|  |  |  |  |  |  |  |  |  |  |
| Genus | CK_D1 | CK_D2 | CK_D3 | E1_D1 | E1_D2 | E1_D3 | E3_D1 | E3_D2 | E3_D3 |
| Fungi_gen_Incertae_sedis | 0.05±0.01b | 0.16±0.12b | 0.17±0.21b | 0.69±0.13a | 0.13±0.04b | 0.18±0.22b | 0.17±0.05b | 0.28±0.05b | 0.19±0.08b |
| *Saitozyma* | 0.22±0.11a | 0.03±0.01c | 0.01±0.01c | 0.00±0.00c | 0.01±0.01c | 0.00±0.00c | 0.09±0.03bc | 0.08±0.06bc | 0.16±0.06ab |
| *Laccaria* | 0.00±0.00b | 0.00±0.00b | 0.00±0.00b | 0.08±0.06ab | 0.23±0.04a | 0.1±0.17ab | 0.11±0.07ab | 0.01±0.02b | 0.01±0.02b |
| *Melanconiella* | 0.01±0.00b | 0.04±0.02b | 0.03±0.04b | 0.01±0.01b | 0.13±0.08a | 0.09±0.05ab | 0.02±0.02b | 0.04±0.03b | 0.01±0.00b |
| *Clitocybula* | 0.00±0.00a | 0.16±0.27a | 0.21±0.35a | 0.00±0.00a | 0.00±0.00a | 0.00±0.00a | 0.00±0.00a | 0.00±0.00a | 0.00±0.00a |
| Thelephoraceae_gen_Incertae_sedis | 0.00±0.00a | 0.00±0.00a | 0.00±0.00a | 0.00±0.00a | 0.07±0.07a | 0.20±0.28a | 0.00±0.00a | 0.04±0.06a | 0.00±0.00a |
| *Sagenomella* | 0.08±0.02ab | 0.08±0.02ab | 0.02±0.03b | 0.00±0.00b | 0.00±0.00b | 0.00±0.00b | 0.00±0.00b | 0.13±0.11a | 0.01±0.00b |
| *Mortierella* | 0.01±0.01b | 0.01±0.01b | 0.03±0.02b | 0.00±0.00b | 0.12±0.05a | 0.01±0.01b | 0.05±0.05b | 0.04±0.02b | 0.02±0.01b |
| *Leohumicola* | 0.04±0.06a | 0.07±0.09a | 0.10±0.15a | 0.00±0.00a | 0.00±0.00a | 0.00±0.00a | 0.01±0.01a | 0.04±0.03a | 0.00±0.00a |
| *Scytinostroma* | 0.00±0.00a | 0.00±0.00a | 0.00±0.00a | 0.00±0.00a | 0.00±0.00a | 0.26±0.45a | 0.00±0.00a | 0.00±0.00a | 0.00±0.00a |

Different lowercase letters indicate significant differences between groups, with *p* < 0.05.
